# Supplementary material for: Analysis of the nischarin expression across human tumor types reveals its context-dependent role and a potential as a target for drug repurposing in oncology
Source: PLoS One. 2024 May 23;19(5):e0299685. doi: 10.1371/journal.pone.0299685 (PMC11115306; doi:10.1371/journal.pone.0299685)
Supplement: S3 Fig — (PDF) [file pone.0299685.s003.pdf]

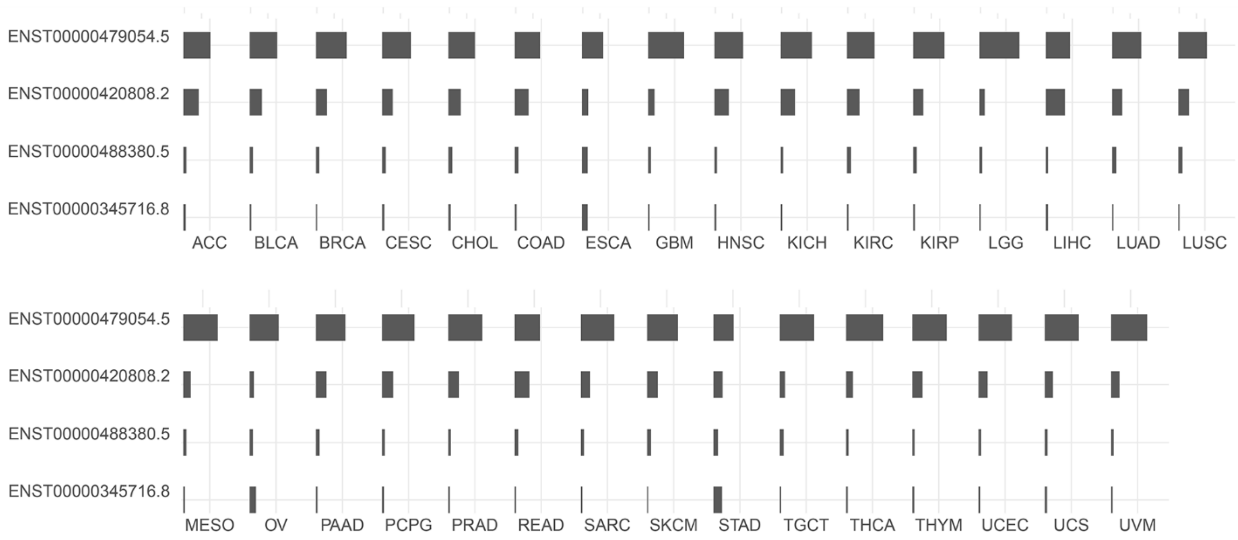


**S3 Fig. NISCH isoform usage distribution across solid TCGA cancers according to the GEPIA2 website.**
