# Supplementary figures and images for: Analysis of the nischarin expression across human tumor types reveals its context-dependent role and a potential as a target for drug repurposing in oncology
Source: PLoS One. 2024 May 23;19(5):e0299685. doi: 10.1371/journal.pone.0299685 (PMC11115306; doi:10.1371/journal.pone.0299685)

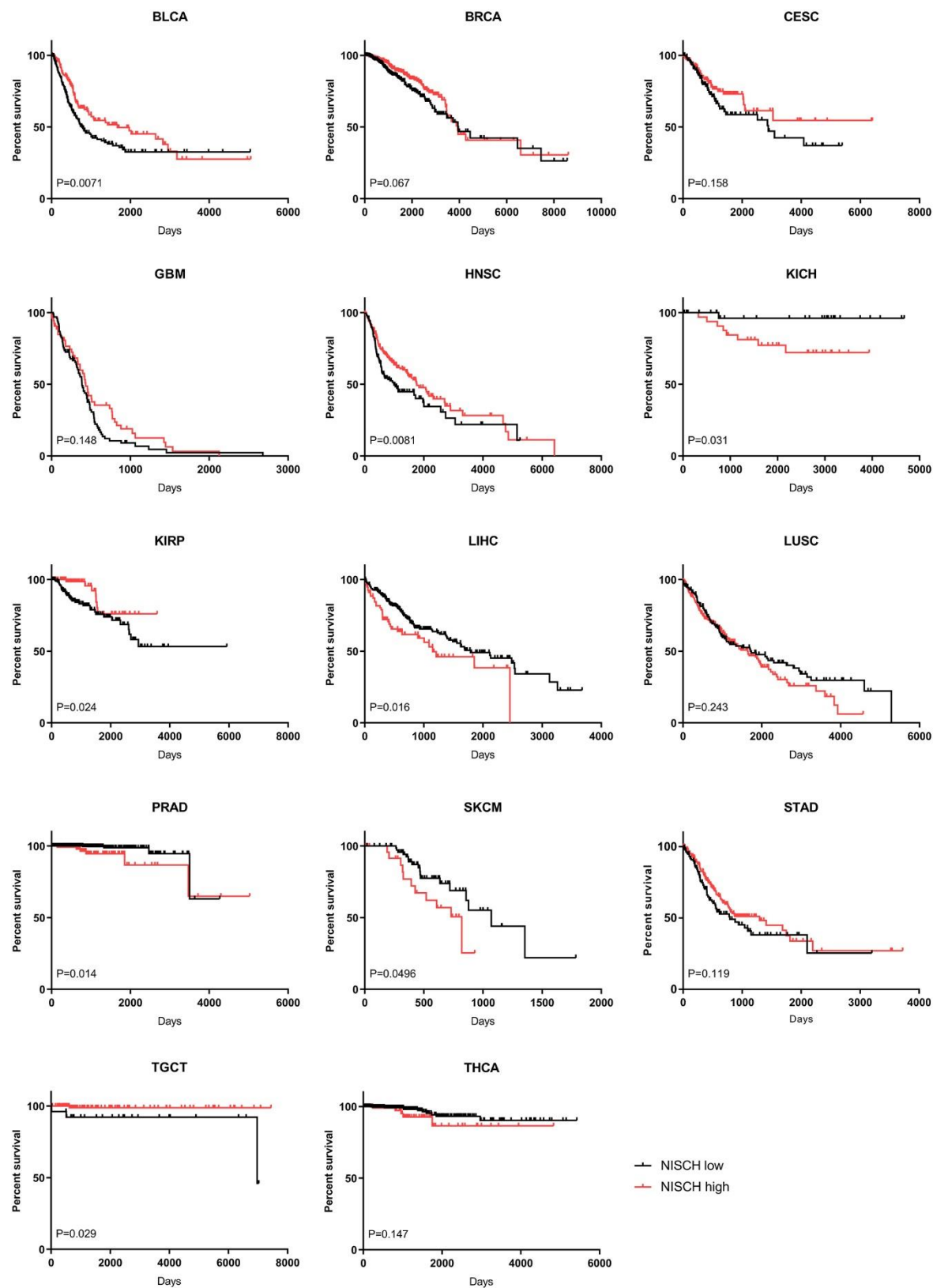

**S4 Fig. NISCH prognostic value across different tumors.**

Supplement: S4 Fig — (PDF) [file pone.0299685.s004.pdf]

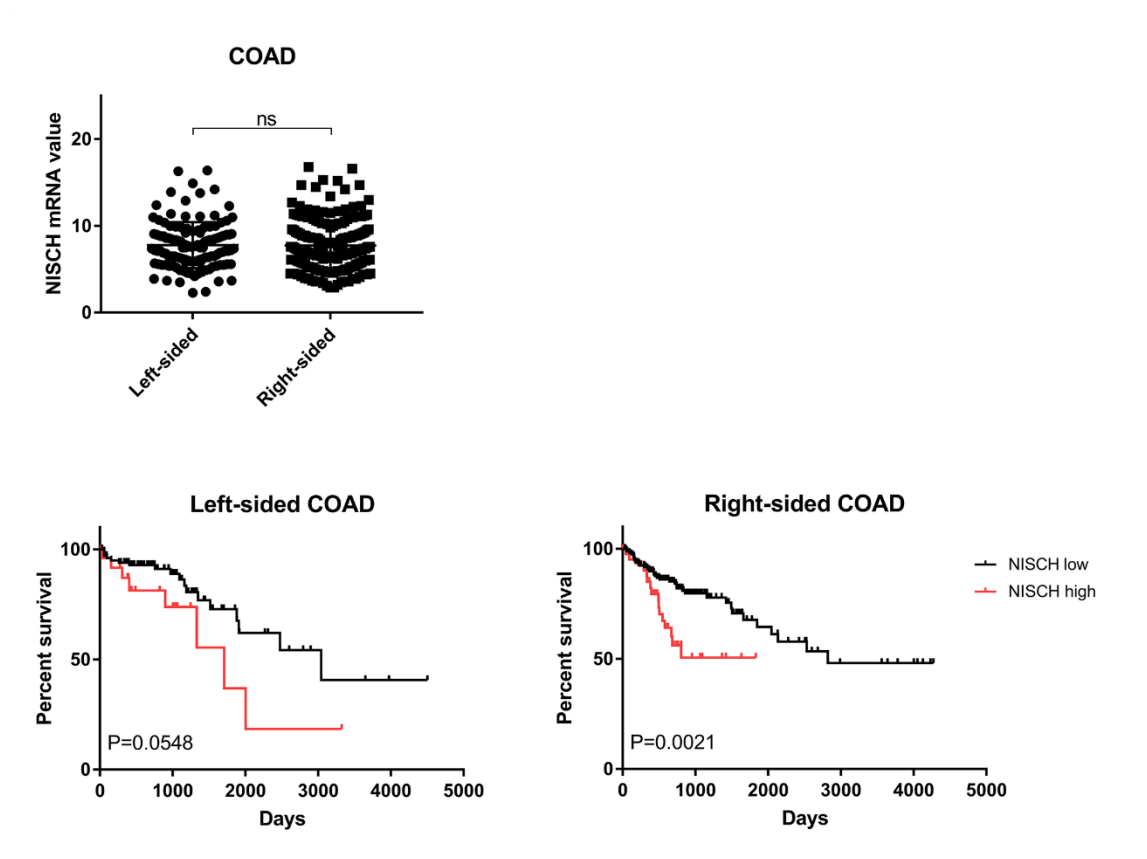

**S5 Fig. *NISCH* mRNA expression differences and Kaplan-Meier plots for left- and right-sided COAD.**

Supplement: S5 Fig — (PDF) [file pone.0299685.s005.pdf]
