# Supplementary material for: Analysis of the nischarin expression across human tumor types reveals its context-dependent role and a potential as a target for drug repurposing in oncology
Source: PLoS One. 2024 May 23;19(5):e0299685. doi: 10.1371/journal.pone.0299685 (PMC11115306; doi:10.1371/journal.pone.0299685)
Supplement: S6 Fig — (PDF) [file pone.0299685.s006.pdf]

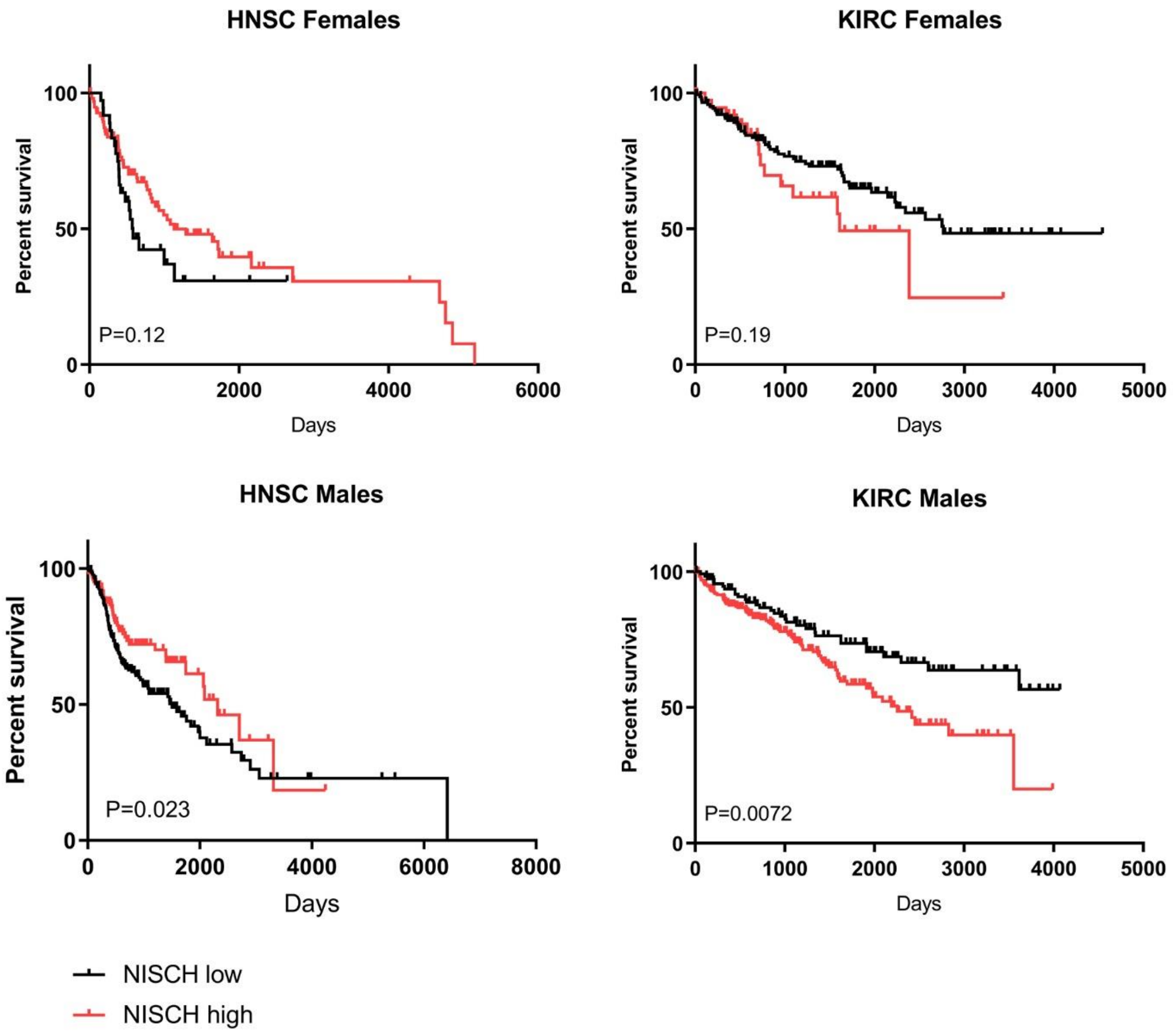

S6 Fig. The effect of nischarin mRNA expression on overall survival of patients of the opposite sex. Primary (**A**) KIRC and (**B**) HNSC tumor samples were divided into high and low NISCH groups for both sexes based on the best *NISCH* expression cut-off that yields maximal survival difference.
