# Supplementary material for: Analysis of the nischarin expression across human tumor types reveals its context-dependent role and a potential as a target for drug repurposing in oncology
Source: PLoS One. 2024 May 23;19(5):e0299685. doi: 10.1371/journal.pone.0299685 (PMC11115306; doi:10.1371/journal.pone.0299685)
Supplement: S8 Fig — (PDF) [file pone.0299685.s008.pdf]

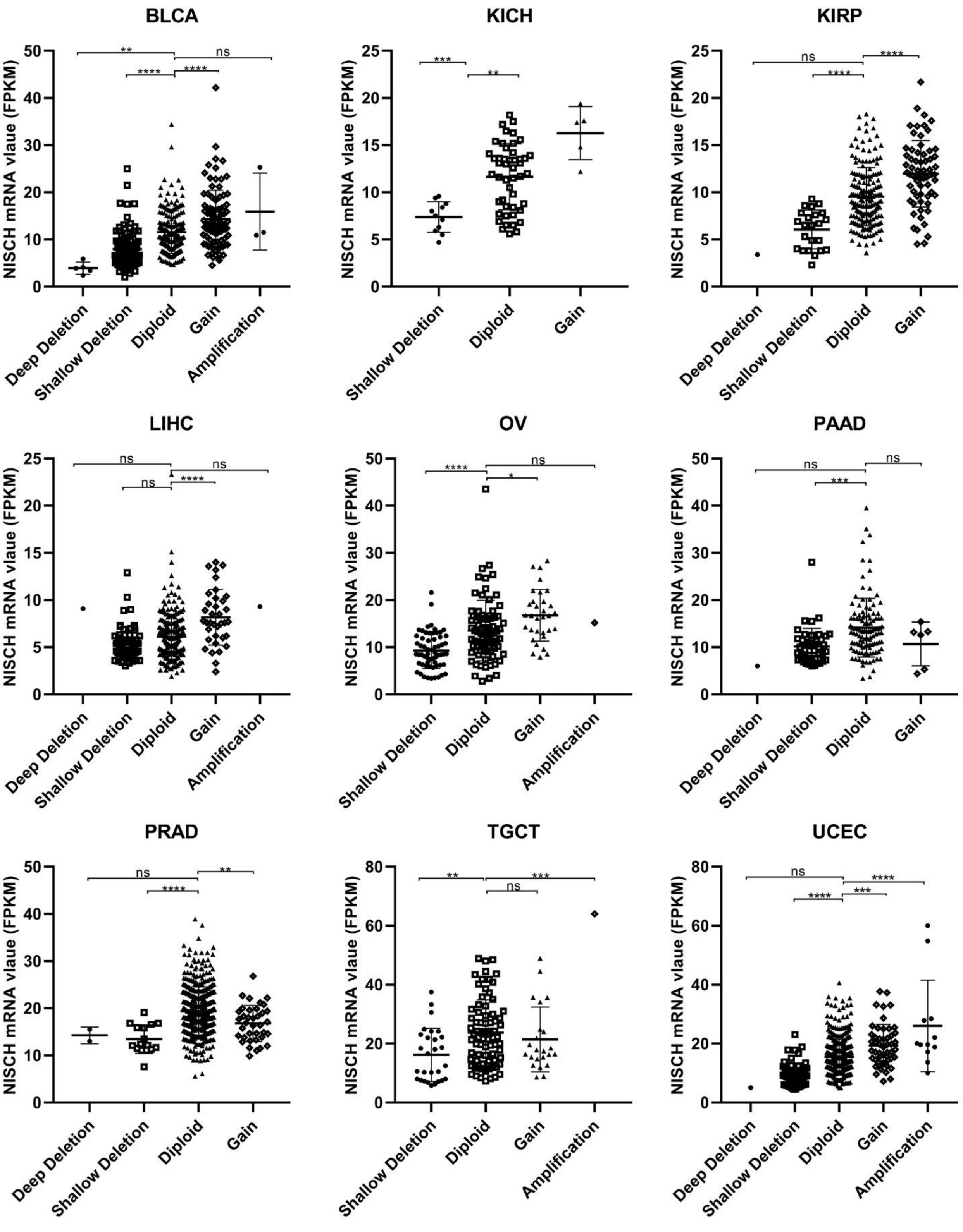

S8 Fig. Copy-number alterations in TCGA cancers in which *NISCH* was identified as prognostic marker. The effect of CNA on *NISCH* mRNA expression.
